# *N*-[2-(*N*-Cyclo­hexyl­carbamo­yl)propan-2-yl]-*N*-(2-iodo­phen­yl)prop-2-ynamide

**DOI:** 10.1107/S1600536811055140

**Published:** 2012-01-07

**Authors:** Saeed Balalaie, Yaghoub Haghighatnia, Frank Rominger, Vahid Amani

**Affiliations:** a Peptide Chemistry Research Center, K. N. Toosi University of Technology, PO Box 15875-4416, Tehran, Iran; b Organisch-Chemisches Institut, Universität Heidelberg, Im Neuenheimer Feld 270, 69120 Heidelberg, Germany; c Shahid Beheshti University, Department of Chemistry, Evin, Tehran 1983963113, Iran

## Abstract

In the title compound, C_19_H_23_IN_2_O_2_, the cyclo­hexane ring adopts a chair conformation, and the mean plane of the propiolamide unit is approximately perpendicular to the benzene ring [dihedral angle = 88.12 (13)°]. Weak intra­molecular C—H⋯O hydrogen bonding is observed between the carbonyl group and the benzene ring. In the crystal, classical N—H⋯O hydrogen bonds and weak C—H⋯O inter­actions are present.

## Related literature

For background to multi-component reactions (MCRs), see: Dömling & Ugi (2000 ▶); Tietze (1996 ▶); Tietze *et al.* (2006 ▶); Dömling (2006 ▶); Zhu & Bienayme (2005 ▶).
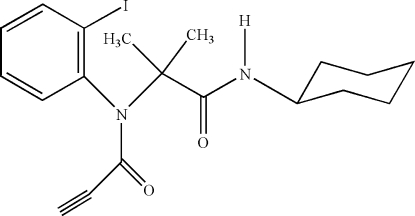


## Experimental

### 

#### Crystal data

C_19_H_23_IN_2_O_2_
*M* *_r_* = 438.29
Orthorhombic, 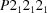

*a* = 7.7511 (3) Å
*b* = 10.0726 (4) Å
*c* = 24.6063 (9) Å
*V* = 1921.11 (13) Å^3^
*Z* = 4
Mo *K*α radiation
μ = 1.68 mm^−1^
*T* = 200 K
0.19 × 0.08 × 0.06 mm

#### Data collection

Bruker APEXII Quazar diffractometer
Absorption correction: multi-scan (*SADABS*; Bruker, 2001 ▶). *T*_min_ = 0.741, *T*_max_ = 0.906
25494 measured reflections
4795 independent reflections
4399 reflections with *I* > 2σ(*I*)
*R*_int_ = 0.024

#### Refinement

*R*[*F* ^2^ > 2σ(*F* ^2^)] = 0.027
*wR*(*F* ^2^) = 0.060
*S* = 1.04
4795 reflections
224 parameters
H atoms treated by a mixture of independent and constrained refinement
Δρ_max_ = 0.83 e Å^−3^
Δρ_min_ = −0.90 e Å^−3^
Absolute structure: Flack (1983 ▶), 2041 Friedel pairs
Flack parameter: 0.237 (16)

### 

Data collection: *APEX2* (Bruker, 2007 ▶); cell refinement: *SAINT* (Bruker, 2007 ▶); data reduction: *SAINT*; program(s) used to solve structure: *SHELXTL* (Sheldrick, 2008 ▶); program(s) used to refine structure: *SHELXTL*; molecular graphics: *SHELXTL*; software used to prepare material for publication: *SHELXTL*.

## Supplementary Material

Crystal structure: contains datablock(s) I, global. DOI: 10.1107/S1600536811055140/xu5415sup1.cif

Structure factors: contains datablock(s) I. DOI: 10.1107/S1600536811055140/xu5415Isup2.hkl

Supplementary material file. DOI: 10.1107/S1600536811055140/xu5415Isup3.cml

Additional supplementary materials:  crystallographic information; 3D view; checkCIF report

## Figures and Tables

**Table 1:** Hydrogen-bond geometry (Å, °)

*D*—H⋯*A*	*D*—H	H⋯*A*	*D*⋯*A*	*D*—H⋯*A*
N1—H1⋯O6^i^	0.79 (3)	2.25 (3)	3.016 (3)	164 (3)
C4—H4*C*⋯O6^i^	0.98	2.42	3.291 (3)	148
C26—H26⋯O1	0.95	2.57	3.270 (3)	131

Symmetry code: (i) 
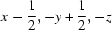
.

**Fig. 1.**
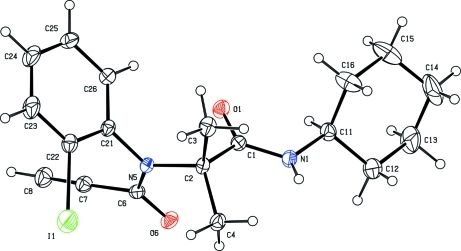
The molecular structure of the title molecule, with the atom-numbering scheme. Displacement ellipsoids are drawn at the 30% probability level.

**Fig. 2.**
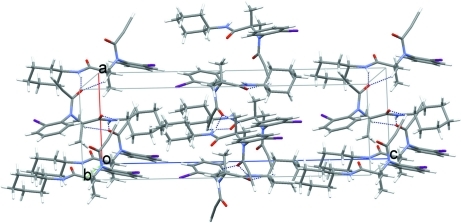
Unit-cell packing diagram for title compound.

## supplementary crystallographic information

### Comment

Multicomponent reactions (MCRs) have attracted considerable interest owing to their exceptional synthetic efficiency (Dömling & Ugi, 2000; Tietze, 1996; Tietze *et al.*, 2006). Especially isocyanide based MCRs (IMCRs) allow for the synthesis of a large number of different scaffolds. The design of novel IMCRs has attracted great attention for construction of different organic functional groups (Dömling, 2006; Zhu & Bienayme, 2005).

The iodophenyl group in the title compound is oriented orthogonal to the amid group, meaning that there is no conjugation between these two pi systems. The two amid groups themselves however are planar as expected, indicating a considerable amount of π- conjugation in the N—C=O units, thus partially double bond character and hindered rotation around the amide single bonds. The crystal lattice is stabilized by weak intermolecular N—H···O=C type hydrogen bonding with N1 acting as hydrogen donor and O6 as hydrogen acceptor, leading to one-dimenssional chains in crystallographic a direction. The N···O distance amounts to 3.015 (3) Å and the N—H···O angle to 163 (1)°. Intermolecular N—H···O and C—H···O hydrogen bond are effective in the stabilization of the crystal structure of the title compound (Table 1 & Fig. 2).

### Experimental

The product was obtained *via* a four-component reaction of acetone, 2-iodo-aniline, propiolic acid, and cyclohexylisocyanide in methanol at room temperature. To a solution of acetone (58 mg, 1 mmole) in methanol (5 mL) 2-iodo-aniline (219 mg, 1 mmol) was added. The reaction mixture was stirred at room temperature for 1 h. Then propiolic acid (70 mg, 1 mmol) was added and stirring was continued for 15 min, followed by the addition of cyclohexylisocyanide (1 mmol, 123 mg). After stirring for 24 h at room temperature the reaction mixture was neutralized with 30 mL saturated aqueous NaHCO_3_ solution and extracted with EtOAc (3 × 20 mL). The combined organic layers were dried with anhydrous magnesium sulfate and the solvent was evaporated. The residue was crystallized from acetonitrile.

### Refinement

Imino H atom was located in a difference Fourier map and refined isotropically. Other H atoms were positioned geometrically with C—H = 0.95–1.00 Å and constrained to ride on their parent atoms, U_iso_(H) = 1.5U_eq_(C) for methyl H atoms and 1.2U_eq_(C) for the others.

### Crystal data

**Table:**

C_19_H_23_IN_2_O_2_	*F*(000) = 880
*M**_r_* = 438.29	*D*_x_ = 1.515 Mg m^−^^3^
Orthorhombic, *P*2_1_2_1_2_1_	Mo *K*α radiation, λ = 0.71073 Å
Hall symbol: P 2ac 2ab	Cell parameters from 9927 reflections
*a* = 7.7511 (3) Å	θ = 2.6–30.2°
*b* = 10.0726 (4) Å	µ = 1.68 mm^−^^1^
*c* = 24.6063 (9) Å	*T* = 200 K
*V* = 1921.11 (13) Å^3^	Polyhedron, colourless
*Z* = 4	0.19 × 0.08 × 0.06 mm

### Data collection

**Table:**

Bruker APEXII Quazar diffractometer	4795 independent reflections
Radiation source: ImuS microsource	4399 reflections with *I* > 2σ(*I*)
mirror	*R*_int_ = 0.024
ω scans	θ_max_ = 28.4°, θ_min_ = 1.7°
Absorption correction: multi-scan (*SADABS*; Bruker, 2001).	*h* = −10→10
*T*_min_ = 0.741, *T*_max_ = 0.906	*k* = −13→13
25494 measured reflections	*l* = −32→32

### Refinement

**Table:**

Refinement on *F*^2^	Secondary atom site location: difference Fourier map
Least-squares matrix: full	Hydrogen site location: inferred from neighbouring sites
*R*[*F*^2^ > 2σ(*F*^2^)] = 0.027	H atoms treated by a mixture of independent and constrained refinement
*wR*(*F*^2^) = 0.060	*w* = 1/[σ^2^(*F*_o_^2^) + (0.0194*P*)^2^ + 1.3105*P*] where *P* = (*F*_o_^2^ + 2*F*_c_^2^)/3
*S* = 1.04	(Δ/σ)_max_ = 0.001
4795 reflections	Δρ_max_ = 0.83 e Å^−^^3^
224 parameters	Δρ_min_ = −0.90 e Å^−^^3^
0 restraints	Absolute structure: Flack (1983), 2041 Friedel pairs
Primary atom site location: structure-invariant direct methods	Flack parameter: 0.237 (16)

### Special details

**Table:**

Experimental. Hydrogen atom positions were calculated according to geometrical criteria except the amide hydrogen atom H1, which was refined isotropically. The thermal parameters of the hydrogen atoms were set to be 1.2 times the *U*_eq_ of the preceding carbon atom, 1.5 for the methyl groups. The conformation of the methyl hydrogen atoms was allowed to refine. The symmetry of the crystal is chiral, albeit a racemic twinning parameter was introduced and refined to 24% racemic twinning.
Geometry. All e.s.d.'s (except the e.s.d. in the dihedral angle between two l.s. planes) are estimated using the full covariance matrix. The cell e.s.d.'s are taken into account individually in the estimation of e.s.d.'s in distances, angles and torsion angles; correlations between e.s.d.'s in cell parameters are only used when they are defined by crystal symmetry. An approximate (isotropic) treatment of cell e.s.d.'s is used for estimating e.s.d.'s involving l.s. planes.
Refinement. Refinement of *F*^2^ against ALL reflections. The weighted *R*-factor *wR* and goodness of fit *S* are based on *F*^2^, conventional *R*-factors *R* are based on *F*, with *F* set to zero for negative *F*^2^. The threshold expression of *F*^2^ > σ(*F*^2^) is used only for calculating *R*-factors(gt) *etc*. and is not relevant to the choice of reflections for refinement. *R*-factors based on *F*^2^ are statistically about twice as large as those based on *F*, and *R*- factors based on ALL data will be even larger.

### Fractional atomic coordinates and isotropic or equivalent isotropic displacement parameters (Å^2^)

**Table:**

	*x*	*y*	*z*	*U*_iso_*/*U*_eq_	
I1	0.57763 (4)	0.484096 (19)	−0.195485 (7)	0.06160 (8)	
C1	0.5745 (3)	0.45305 (19)	0.02668 (8)	0.0263 (4)	
O1	0.6285 (2)	0.55977 (16)	0.04340 (7)	0.0345 (4)	
N1	0.5421 (3)	0.3503 (2)	0.05975 (8)	0.0309 (4)	
H1	0.500 (4)	0.286 (3)	0.0467 (12)	0.040 (9)*	
C2	0.5255 (3)	0.4367 (2)	−0.03397 (9)	0.0267 (5)	
C3	0.3395 (3)	0.4863 (3)	−0.03893 (10)	0.0359 (5)	
H3A	0.3070	0.4905	−0.0774	0.054*	
H3B	0.3303	0.5749	−0.0228	0.054*	
H3C	0.2621	0.4251	−0.0198	0.054*	
C4	0.5371 (3)	0.2937 (2)	−0.05554 (10)	0.0327 (6)	
H4A	0.6521	0.2578	−0.0480	0.049*	
H4B	0.5167	0.2935	−0.0949	0.049*	
H4C	0.4498	0.2387	−0.0376	0.049*	
N5	0.6435 (2)	0.52550 (19)	−0.06570 (7)	0.0235 (3)	
C6	0.8158 (3)	0.5074 (2)	−0.05990 (8)	0.0260 (4)	
O6	0.8799 (2)	0.41486 (16)	−0.03473 (7)	0.0335 (4)	
C7	0.9261 (3)	0.6074 (2)	−0.08547 (9)	0.0293 (4)	
C8	1.0242 (3)	0.6882 (3)	−0.10125 (12)	0.0403 (6)	
H8	1.1032	0.7533	−0.1140	0.048*	
C11	0.5534 (3)	0.3660 (2)	0.11881 (9)	0.0322 (5)	
H11	0.6491	0.4301	0.1262	0.039*	
C12	0.6003 (6)	0.2373 (3)	0.14650 (11)	0.0576 (9)	
H12A	0.7101	0.2031	0.1314	0.069*	
H12B	0.5093	0.1704	0.1397	0.069*	
C13	0.6195 (6)	0.2595 (4)	0.20757 (12)	0.0733 (12)	
H13A	0.6457	0.1739	0.2255	0.088*	
H13B	0.7172	0.3206	0.2144	0.088*	
C14	0.4561 (7)	0.3178 (5)	0.23188 (13)	0.0853 (15)	
H14A	0.4738	0.3351	0.2711	0.102*	
H14B	0.3602	0.2535	0.2281	0.102*	
C15	0.4094 (6)	0.4470 (4)	0.20299 (14)	0.0775 (11)	
H15A	0.5001	0.5140	0.2100	0.093*	
H15B	0.2994	0.4814	0.2178	0.093*	
C16	0.3911 (4)	0.4258 (4)	0.14159 (13)	0.0591 (9)	
H16A	0.2924	0.3662	0.1342	0.071*	
H16B	0.3681	0.5120	0.1236	0.071*	
C21	0.5826 (3)	0.6458 (2)	−0.09059 (9)	0.0260 (4)	
C22	0.5475 (3)	0.6514 (2)	−0.14595 (10)	0.0330 (5)	
C23	0.4942 (4)	0.7698 (3)	−0.16949 (12)	0.0459 (7)	
H23	0.4712	0.7737	−0.2074	0.055*	
C24	0.4746 (4)	0.8817 (3)	−0.13790 (13)	0.0481 (8)	
H24	0.4374	0.9624	−0.1541	0.058*	
C25	0.5086 (3)	0.8773 (3)	−0.08294 (13)	0.0430 (6)	
H25	0.4946	0.9547	−0.0613	0.052*	
C26	0.5634 (4)	0.7595 (2)	−0.05932 (10)	0.0331 (5)	
H26	0.5880	0.7566	−0.0215	0.040*	

### Atomic displacement parameters (Å^2^)

**Table:**

	*U*^11^	*U*^22^	*U*^33^	*U*^12^	*U*^13^	*U*^23^
I1	0.10850 (18)	0.04834 (10)	0.02796 (8)	−0.00472 (12)	−0.00363 (10)	−0.00355 (8)
C1	0.0263 (10)	0.0266 (10)	0.0261 (9)	0.0010 (9)	0.0000 (9)	0.0007 (7)
O1	0.0449 (10)	0.0277 (8)	0.0308 (8)	−0.0074 (7)	−0.0019 (7)	−0.0011 (7)
N1	0.0416 (12)	0.0262 (9)	0.0248 (9)	−0.0054 (9)	0.0019 (9)	0.0003 (7)
C2	0.0308 (12)	0.0244 (10)	0.0250 (11)	−0.0029 (8)	−0.0007 (8)	0.0048 (9)
C3	0.0266 (11)	0.0392 (14)	0.0420 (13)	−0.0034 (11)	−0.0014 (9)	0.0066 (13)
C4	0.0435 (15)	0.0254 (11)	0.0294 (12)	−0.0066 (10)	−0.0008 (11)	0.0001 (9)
N5	0.0254 (8)	0.0219 (8)	0.0233 (8)	0.0010 (7)	−0.0015 (6)	0.0034 (7)
C6	0.0282 (10)	0.0234 (11)	0.0264 (9)	0.0031 (9)	0.0002 (8)	−0.0019 (9)
O6	0.0319 (9)	0.0286 (8)	0.0401 (9)	0.0064 (7)	−0.0021 (7)	0.0075 (7)
C7	0.0265 (10)	0.0293 (10)	0.0320 (10)	0.0021 (10)	−0.0015 (11)	0.0016 (8)
C8	0.0374 (14)	0.0369 (14)	0.0465 (16)	−0.0044 (11)	0.0019 (11)	0.0054 (11)
C11	0.0388 (13)	0.0325 (11)	0.0252 (11)	−0.0060 (11)	−0.0009 (10)	−0.0008 (8)
C12	0.093 (3)	0.0495 (16)	0.0300 (13)	0.0162 (19)	0.0072 (17)	0.0079 (12)
C13	0.117 (4)	0.071 (2)	0.0311 (16)	0.002 (2)	−0.0048 (18)	0.0153 (14)
C14	0.124 (4)	0.104 (3)	0.0283 (15)	−0.038 (3)	0.023 (2)	−0.0124 (18)
C15	0.081 (2)	0.101 (3)	0.0510 (19)	0.007 (2)	0.017 (2)	−0.0330 (19)
C16	0.0514 (19)	0.079 (2)	0.0472 (17)	0.0156 (17)	0.0047 (14)	−0.0178 (16)
C21	0.0223 (9)	0.0254 (10)	0.0303 (10)	0.0006 (9)	−0.0009 (10)	0.0064 (8)
C22	0.0331 (13)	0.0351 (12)	0.0308 (12)	0.0005 (10)	−0.0031 (10)	0.0072 (9)
C23	0.0486 (16)	0.0490 (17)	0.0401 (15)	0.0054 (13)	−0.0056 (12)	0.0194 (13)
C24	0.0412 (15)	0.0388 (15)	0.0643 (19)	0.0131 (12)	0.0040 (13)	0.0244 (14)
C25	0.0442 (14)	0.0277 (12)	0.0572 (17)	0.0095 (11)	0.0136 (13)	0.0051 (12)
C26	0.0350 (13)	0.0285 (11)	0.0356 (12)	0.0018 (11)	0.0042 (12)	0.0025 (9)

### Geometric parameters (Å, °)

**Table:**

I1—C22	2.093 (3)	C12—C13	1.526 (4)
C1—O1	1.225 (3)	C12—H12A	0.9900
C1—N1	1.341 (3)	C12—H12B	0.9900
C1—C2	1.549 (3)	C13—C14	1.519 (6)
N1—C11	1.464 (3)	C13—H13A	0.9900
N1—H1	0.80 (3)	C13—H13B	0.9900
C2—N5	1.499 (3)	C14—C15	1.526 (6)
C2—C3	1.531 (3)	C14—H14A	0.9900
C2—C4	1.538 (3)	C14—H14B	0.9900
C3—H3A	0.9800	C15—C16	1.532 (4)
C3—H3B	0.9800	C15—H15A	0.9900
C3—H3C	0.9800	C15—H15B	0.9900
C4—H4A	0.9800	C16—H16A	0.9900
C4—H4B	0.9800	C16—H16B	0.9900
C4—H4C	0.9800	C21—C26	1.387 (3)
N5—C6	1.356 (3)	C21—C22	1.390 (3)
N5—C21	1.438 (3)	C22—C23	1.389 (4)
C6—O6	1.225 (3)	C23—C24	1.377 (4)
C6—C7	1.463 (3)	C23—H23	0.9500
C7—C8	1.179 (3)	C24—C25	1.379 (4)
C8—H8	0.9500	C24—H24	0.9500
C11—C16	1.503 (4)	C25—C26	1.388 (3)
C11—C12	1.509 (4)	C25—H25	0.9500
C11—H11	1.0000	C26—H26	0.9500
			
O1—C1—N1	122.5 (2)	H12A—C12—H12B	108.2
O1—C1—C2	120.06 (19)	C14—C13—C12	111.3 (3)
N1—C1—C2	117.20 (19)	C14—C13—H13A	109.4
C1—N1—C11	120.5 (2)	C12—C13—H13A	109.4
C1—N1—H1	117 (2)	C14—C13—H13B	109.4
C11—N1—H1	121 (2)	C12—C13—H13B	109.4
N5—C2—C3	109.78 (17)	H13A—C13—H13B	108.0
N5—C2—C4	110.11 (19)	C13—C14—C15	110.1 (3)
C3—C2—C4	109.4 (2)	C13—C14—H14A	109.6
N5—C2—C1	106.81 (17)	C15—C14—H14A	109.6
C3—C2—C1	105.83 (19)	C13—C14—H14B	109.6
C4—C2—C1	114.71 (19)	C15—C14—H14B	109.6
C2—C3—H3A	109.5	H14A—C14—H14B	108.2
C2—C3—H3B	109.5	C14—C15—C16	111.3 (3)
H3A—C3—H3B	109.5	C14—C15—H15A	109.4
C2—C3—H3C	109.5	C16—C15—H15A	109.4
H3A—C3—H3C	109.5	C14—C15—H15B	109.4
H3B—C3—H3C	109.5	C16—C15—H15B	109.4
C2—C4—H4A	109.5	H15A—C15—H15B	108.0
C2—C4—H4B	109.5	C11—C16—C15	110.3 (3)
H4A—C4—H4B	109.5	C11—C16—H16A	109.6
C2—C4—H4C	109.5	C15—C16—H16A	109.6
H4A—C4—H4C	109.5	C11—C16—H16B	109.6
H4B—C4—H4C	109.5	C15—C16—H16B	109.6
C6—N5—C21	118.79 (19)	H16A—C16—H16B	108.1
C6—N5—C2	117.79 (19)	C26—C21—C22	119.3 (2)
C21—N5—C2	121.66 (17)	C26—C21—N5	119.6 (2)
O6—C6—N5	123.7 (2)	C22—C21—N5	121.0 (2)
O6—C6—C7	120.31 (19)	C23—C22—C21	120.1 (2)
N5—C6—C7	116.0 (2)	C23—C22—I1	118.8 (2)
C8—C7—C6	173.3 (3)	C21—C22—I1	121.10 (17)
C7—C8—H8	180.0	C24—C23—C22	120.0 (3)
N1—C11—C16	111.3 (2)	C24—C23—H23	120.0
N1—C11—C12	111.7 (2)	C22—C23—H23	120.0
C16—C11—C12	112.2 (3)	C23—C24—C25	120.4 (2)
N1—C11—H11	107.1	C23—C24—H24	119.8
C16—C11—H11	107.1	C25—C24—H24	119.8
C12—C11—H11	107.1	C24—C25—C26	119.8 (3)
C11—C12—C13	110.0 (3)	C24—C25—H25	120.1
C11—C12—H12A	109.7	C26—C25—H25	120.1
C13—C12—H12A	109.7	C21—C26—C25	120.4 (2)
C11—C12—H12B	109.7	C21—C26—H26	119.8
C13—C12—H12B	109.7	C25—C26—H26	119.8
			
O1—C1—N1—C11	6.4 (4)	C11—C12—C13—C14	56.8 (4)
C2—C1—N1—C11	−168.3 (2)	C12—C13—C14—C15	−56.6 (4)
O1—C1—C2—N5	32.0 (3)	C13—C14—C15—C16	56.0 (5)
N1—C1—C2—N5	−153.1 (2)	N1—C11—C16—C15	−177.5 (3)
O1—C1—C2—C3	−84.9 (3)	C12—C11—C16—C15	56.5 (4)
N1—C1—C2—C3	90.0 (2)	C14—C15—C16—C11	−55.7 (5)
O1—C1—C2—C4	154.3 (2)	C6—N5—C21—C26	−84.0 (3)
N1—C1—C2—C4	−30.8 (3)	C2—N5—C21—C26	80.6 (3)
C3—C2—N5—C6	171.13 (19)	C6—N5—C21—C22	94.0 (3)
C4—C2—N5—C6	−68.3 (3)	C2—N5—C21—C22	−101.4 (3)
C1—C2—N5—C6	56.8 (2)	C26—C21—C22—C23	0.1 (4)
C3—C2—N5—C21	6.5 (3)	N5—C21—C22—C23	−177.9 (2)
C4—C2—N5—C21	127.0 (2)	C26—C21—C22—I1	179.06 (19)
C1—C2—N5—C21	−107.8 (2)	N5—C21—C22—I1	1.1 (3)
C21—N5—C6—O6	172.8 (2)	C21—C22—C23—C24	−0.5 (4)
C2—N5—C6—O6	7.7 (3)	I1—C22—C23—C24	−179.5 (2)
C21—N5—C6—C7	−6.7 (3)	C22—C23—C24—C25	0.4 (4)
C2—N5—C6—C7	−171.79 (19)	C23—C24—C25—C26	0.2 (4)
C1—N1—C11—C16	82.0 (3)	C22—C21—C26—C25	0.5 (4)
C1—N1—C11—C12	−151.7 (3)	N5—C21—C26—C25	178.5 (2)
N1—C11—C12—C13	177.2 (3)	C24—C25—C26—C21	−0.6 (4)
C16—C11—C12—C13	−57.0 (4)		

### Hydrogen-bond geometry (Å, °)

**Table:**

*D*—H···*A*	*D*—H	H···*A*	*D*···*A*	*D*—H···*A*
N1—H1···O6^i^	0.79 (3)	2.25 (3)	3.016 (3)	164 (3)
C4—H4C···O6^i^	0.98	2.42	3.291 (3)	148
C26—H26···O1	0.95	2.57	3.270 (3)	131

Symmetry codes: (i) *x*−1/2, −*y*+1/2, −*z*.
